# Adverse childhood experiences and life opportunities: Shifting the narrative

**DOI:** 10.1016/j.childyouth.2016.10.021

**Published:** 2017-01-14

**Authors:** Marilyn Metzler, Melissa T. Merrick, Joanne Klevens, Katie A. Ports, Derek C. Ford

**Affiliations:** a Division of Violence Prevention, Centers for Disease Control and Prevention, Atlanta, GA; b Karna, LLC, Atlanta, GA

**Keywords:** Adverse Childhood Experiences, early adversity, child abuse and neglect, life potential, intergenerational poverty

## Abstract

Substantial research shows that early adversity, including child abuse and neglect, is associated with diminished health across the life course and across generations. Less well understood is the relationship between early adversity and adult socioeconomic status, including education, employment, and income. Collectively, these outcomes provide an indication of overall life opportunity. We analyzed data from 10 states and the District of Columbia that used the adverse childhood experiences (ACE) module in the 2010 Behavioral Risk Factor Surveillance System to examine the association between ACEs and adult education, employment, and income. Compared to participants with no ACEs, those with higher ACE scores were more likely to report high school non-completion, unemployment, and living in a household below the federal poverty level. This evidence suggests that preventing early adversity may impact health and life opportunities that reverberate across generations. Current efforts to prevent early adversity might be more successful if they broaden public and professional understanding (i.e., the narrative) of the links between early adversity and poverty. We discuss our findings within the context of structural policies and processes that may further contribute to the intergenerational continuity of child abuse and neglect and poverty.

## 1. Introduction

Assuring the healthy development of all children is essential for societies seeking to achieve their full health, social, and economic potential. Preventing early adversity, including child abuse and neglect, is critical if these goals are to be met. Families, communities, organizations, and governments—all of society—must be involved in order to achieve these goals. While all are responsible, some have unique roles. Child protection, for example, is responsible for identifying children and families at risk and providing services to minimize harm and treat trauma when harm has occurred. Public health, on the other hand, is responsible for promoting, protecting, improving, and, when necessary, restoring the health of all people (Last, 2007), which requires understanding, at a population level, why some children and families are at greater risk than others and intervening to promote conditions that reduce or eliminate risk. Given the vast problem that is early adversity and its countless ill effects over the life course and across generations, multiple partnerships and disciplines are vital in creating a shared vision for the health and prosperity of our most vulnerable citizens.

The known associations between early adversity and subsequent adverse outcomes are substantial. In addition to the lifetime economic burden of child abuse and neglect (Fang, Brown, Florence, & Mercy, 2012), decades of research also find a robust, dose-response relationship between child abuse and neglect and other forms of adverse childhood experiences (ACEs), and leading causes of adult morbidity and mortality (Felitti et al., 1998; Gilbert et al., 2010). Previous research has examined the relationship between ACEs and health outcomes, often by controlling for socioeconomic indicators such as education, employment, and income. However, less attention has been paid to early experiences as potential determinants of life opportunities, such as later education, employment, and earning outcomes. Understanding the full impact of early adversity across the life course is important if we are to interrupt the cycle of early adversity across generations and assure that all children and societies reach their full health and human potential.

### 1.1. ACEs and Impact on Health

The original ACE Study, a collaboration between the Centers for Disease Control and Prevention (CDC) and Kaiser Permanente, examined the ACE categories of childhood physical, sexual, and emotional abuse, childhood physical and emotional neglect, witnessing domestic violence as a child, and living with a substance abusing, mentally ill, or incarcerated household member as a child (Felitti et al., 1998). More contemporary ACE-related studies have broadened the construct of early adversity to be even more comprehensive, including sibling and peer victimization, property crimes, and parental death as a child (Finkelhor, Shattuck, Turner, & Hamby, 2013). Irrespective of the particular forms of early adversity examined, the link between experiences in childhood and adolescence and subsequent adult health and well-being has been repeatedly established.

Health conditions and indicators associated with early life adversity include: chronic disease (Felitti et al., 1998; Gilbert et al., 2010); cancer (Brown et al., 2010); sexually transmitted diseases (Felitti et al., 1998); frequent mental distress (Gilbert et al., 2010) and depression (Chapman et al., 2004; Anda et al., 2002); intimate partner violence (Whitfield, Anda, Dube, & Felitti, 2003); suicide attempts (Dube et al., 2001); health risk behaviors such as smoking (Felitti et al., 1998; Ford et al., 2001), alcohol abuse (Dube, Anda, Felitti, Edwards, & Croft, 2002), substance abuse (Dube et al., 2003), sexual risk-taking (Hillis, Anda, Felitti, & Marchbanks, 2001), and youth violence (Fox, Perez, Cass, Baglivio, & Epps, 2015); and increased risk for premature mortality by as many as 19 years (Brown et al., 2009). A dose-response relationship between early adversity and poor health has been observed among adolescents as young as age 14 (Flaherty et al., 2013), and modest associations with health have been observed as early as ages 4 to 6 (Flaherty et al., 2006).

The numerous health outcomes associated with ACEs have been largely explained by neurobiological factors that impact early brain development (McCrory, De Brito, & Viding, 2011; Shonkoff, Boyce, & McEwen, 2009; Shonkoff & Garner, 2012; Danese et al., 2008), the immune system (Bierhaus et al., 2003), and the endocrine system (Colborn, 2004; Roth, Lubin, Funk, & Sweatt, 2009; Szyf, 2009). For example, exposure to chronic stress can induce changes in the architecture of different regions of the developing brain (e.g., amygdala, hippocampus), which can impact a range of important functions, such as regulating the stress response, attention, memory, planning, and learning new skills, and also contribute to dysregulation of inflammatory response systems that can lead to a chronic “wear and tear” effect on multiple organ systems (Shonkoff & Garner, 2012).

In addition to describing the relationships between ACEs and health outcomes, and the likely impacts and processes implicated in such, some researchers also attempt to identify populations most at risk (Centers for Disease Control and Prevention, 2010; Ye & Reyes-Salvail, 2014; Andersen & Blosnich, 2013). For example, Ye and Reyes-Salvail report the distribution of risk whereby they highlight that individuals with low education or low income are more likely to report ACEs and more likely to have ill health effects (Ye & Reyes-Salvail, 2014). In these analyses, consistent gradient patterns are observed, with people with lower education and lower income reporting higher ACEs and those with higher education and higher income reporting fewer ACEs. While the distribution of risk is important, it does not address the temporal direction of the relationship between early adversity on these socioeconomic indicators as *outcomes*. Given that ACEs, by definition, occur in childhood and therefore precede educational attainment, employment, and income, it is likely that ACEs also impact these outcomes, in addition to their impact on health. Thus, these indicators of adult socioeconomic status warrant examination as outcomes of interest in order to expose the relationships between early experiences and subsequent life opportunities.

### 1.2. Early adversity and impact on socioeconomic status

Education, employment, and income are commonly used measures of socioeconomic status in U.S. health research, and each independently and consistently correlates with health (Braveman, Cubbin, Egerter, Williams, & Pamuk, 2010). A small but growing body of research connects child abuse and neglect to later life education, employment, and income. For example, multiple types of child abuse have been shown to negatively impact adult employment status, (Sansone, Leung, & Wiederman, 2012; Zielinski, 2009), and have also been linked to poverty and Medicaid usage (Zielinski, 2009). Adults reporting histories of child abuse and neglect have been shown to have lower levels of education, lower employment earnings, and fewer assets compared to matched controls (Currie & Widom, 2010). Adolescents exposed to violence are at increased risk of lower educational attainment and lower adult employment and income (Covey, Menard, & Franzese, 2013; Macmillan & Hagan, 2004). While these studies are informative, many have limited generalizability because of their highly specific samples (e.g., Sansone et al., 2012) or their scope of exposure to early adversity being limited to child abuse and neglect (e.g., Sansone et al., 2012, Currie & Widom, 2010) or violence only (e.g., Covey et al., 2013, Macmillan & Hagan, 2004).

As noted above, an extensive body of literature has identified associations between additional early adversities, including household substance abuse and parental incarceration, and adult health outcomes. We also know from the ACE Study that many early adversities, beyond child abuse and neglect alone, are common (Felitti et al., 1998). While the ACE questionnaire does not provide an exhaustive list of adversities to which a child could be exposed (e.g., bullying, neighborhood violence) (Finkelhor et al., 2013), it does include additional adversities occurring in the home prior to age 18 and gives us a broader understanding of the impact of early experiences on health. Data demonstrating the link between ACEs and other socioeconomic outcomes, including adult education, employment, or income, is sparse, though dose-response relationships between ACEs and adult employment status (Liu, Croft, Chapman, et al., 2013), and adult work performance and financial stress (Anda et al., 2004) have been documented. Such examinations expand our understanding of the impact of early adversity on multiple outcomes that likely contribute to one’s later socioeconomic status. Recently, Font and Maguire-Jack (Font & Maguire-Jack, 2015) examined and found a mediational role for education, income, and health insurance status in the relationship between ACEs and health. Such methodologically rigorous analyses move the field forward by considering the important explanatory contributions of indicators of socioeconomic status in predicting health outcomes. However, also needed are analyses of early adversity and indicators of socioeconomic status that are explored as separate but connected outcomes of interest.

## 2. Theory

This study was informed by current theories from social epidemiology (Berkman, Kawachi, & Glymour, 2014) around the social construction of health that seek not only to document health outcomes, including child abuse and neglect, but to also examine the social and economic contexts that may contribute to the differential distribution of outcomes. These theories, discussed below, are not mutually exclusive and provide important guidance for understanding the differential burden and impact of early adversity across the life course, which is critical if we are to achieve our U.S. goal to eliminate health inequities (Healthy People 2020). Health inequities are broadly understood as the persistent observation of health and disease, including violence, along social and economic hierarchies including race, ethnicity, class, and gender (Braveman, 2014).

Growing interest in the social determinants of health has led to increased understanding that no single theory can fully explain the complexity of pathways and relationships that may give rise to these inequities. Rather, multiple theories are needed to explain how, for example, health behaviors contributing to poor outcomes are patterned by social and economic conditions. In other words, the choices a person makes are shaped by the choices a person has, which are themselves shaped by structural policies and processes. For example, the ability to live in a safe neighborhood may be limited by housing and economic development policies that locate sidewalks, street lights, and low-traffic streets in neighborhoods with more expensive homes compared to neighborhoods with more affordable homes.

**Fig. 1.**
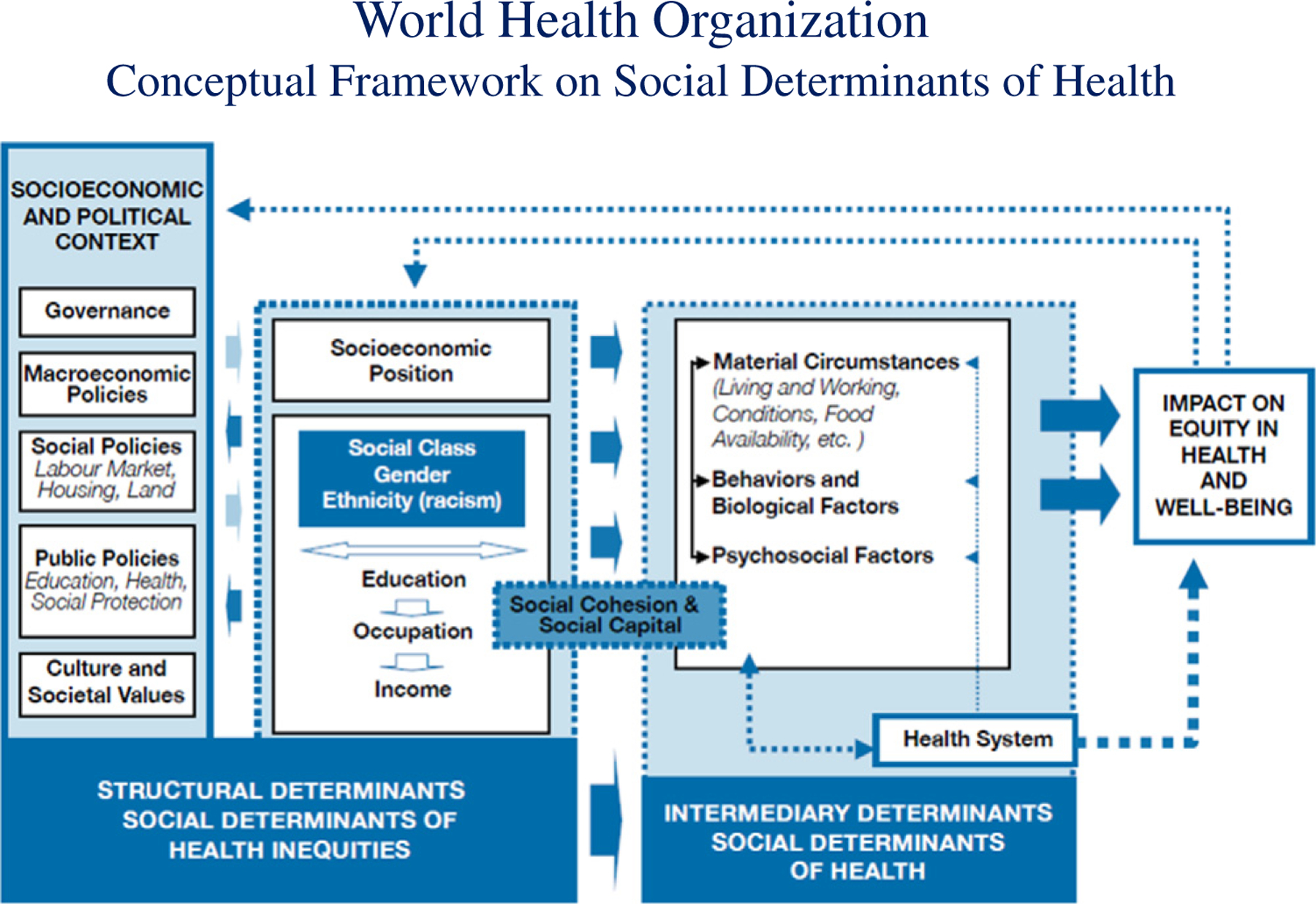
Solar and Irwin, (2010). A conceptual framework for action on the social determinants of health. Geneva: World Health Organization.

To support understanding of how structural determinants contribute to health inequities, the World Health Organization’s Commission on Social Determinants of Health (CSDH) developed a conceptual framework that encompasses multiple, interacting theories (Solar & Irwin, 2010) (see Fig. 1). These include psychosocial theories that focus on people’s perceptions and experiences of being in hierarchies and living in social settings of inequality (Wilkinson & Pickett, 2006); economic and political theories that focus on the impact of structural inequalities on health and disease (Kaplan, Pamuk, Lynch, Cohen, & Balfour, 1996; Smith & Egger, 1996); and ecosocial theories that seek to integrate social, biological, historical, and ecological perspectives in order to develop new insights into determinants of population distribution of disease and social inequities in health (Krieger, 2001). The CSDH framework draws on many models that preceded it, but provides needed specificity to inform in-depth explorations of the mechanisms and pathways through which structural policies and processes contribute to differential exposure, differential vulnerability, and, consequently, differential health outcomes. See Appendix A for additional information about the CSDH framework.

The CSDH framework seeks to explain how the differential impact of structural policies and processes influence socioeconomic position based on race, ethnicity, sex, and other social categories, and how this positioning creates vulnerability through more or less access to living and working conditions needed for health. An understanding of the contribution of structural determinants is needed in order to set reasonable expectations for outcomes. For example, interventions addressing intermediary determinants may improve the situations of those currently living in vulnerable conditions. However, addressing the structural determinants that give rise to these conditions in the first place is necessary to assure equitable, sustainable opportunities for health and safety over the life course and across generations. Finally, and importantly, the framework accounts for human agency in the generation of structures, policies, and processes that create and distribute life chances and opportunities for health by emphasizing the need to include groups historically and currently excluded from societal decision-making processes that impact their health and life opportunities. The distinction between the determinants (e.g., macro-level policies) and the processes that give rise to their distribution (e.g., social and political power) is critical for the development of effective actions to eliminate health inequities. In this paper, the CSDH framework is used to situate the study findings within a larger context in order to increase understanding about why we may be seeing these outcomes and therefore how to more effectively address them.

### 2.1. The present study

The present study examines the impact of ACEs on adult education, employment, and income. Given the persistent observation of the impact of ACEs on multiple health outcomes, we hypothesized that early adversity would increase the likelihood of reduced education, unemployment, and low income. We explore our findings within a larger context to understand socioeconomic status as more than the attributes of individuals, but a consequence of early experiences, and we raise questions about what this means in terms of the current narrative around the intergenerational cycle of early adversity and subsequent impacts on health and life opportunities.

## 3. Material and methods

### 3.1. Participants

Participants for this study consisted of 27,834 noninstitutionalized adults surveyed during the 2010 Behavioral Risk Factor Surveillance System (BRFSS) data collection year in 10 states and the District of Columbia that used the optional ACE module. The BRFSS, coordinated by CDC, is a nationwide, state-operated, cross-sectional, random-digit-dial telephone survey that collects data from noninstitutionalized U.S. adults regarding health conditions and risk factors. The BRFSS uses a complex sampling design that employs survey weights to adjust for nonresponse and noncoverage biases. This weight, along with stratum and primary sampling unit variables to account for clustering, was included in all analyses.

Participants were residents of the District of Columbia or one of the following 10 states: Hawaii, Maine, Nebraska, Nevada, Ohio, Pennsylvania, Utah, Washington, Wisconsin, or Vermont. The final weighted study sample was 84.9% white (95% CI [84.0, 85.7]); 4.7% black (95% CI [4.2, 5.3]); 3.9% Latino (95% CI [3.51, 4.37]); 2.9% Asian (95% CI [2.5, 3.4]); and 3.6% other ethnicities (95% CI [3.2, 3.9]). Ages of the respondents ranged from 18 to 99 years with a mean age of 43.3 years (SE = 0.15); 45.4% of the sample were female, 95% CI [44.2, 46.6].

### 3.2. Measures

We use the following measures in the BRFSS to examine the impact of ACEs on adult education, employment, and income:

#### 3.2.1. BRFSS ACE Module

The BRFSS ACE module asked adults about the following childhood experiences: three types of child abuse (physical, emotional, or sexual) by a parent or other adult in the household; parents separated or divorced; living with parents or adults who were physically violent to each other; and, living with anyone who was depressed, mentally ill, or suicidal, a problem drinker or alcoholic, used illegal street drugs or abused prescription medications, or, served time or was sentenced to serve time in a prison, jail, or other correctional facility. The BRFSS ACE questions are adapted from those in the original ACE study (Felitti et al., 1998) but do not include questions pertaining to neglect. An ACE score was calculated for each participant by summing the total number of reported ACE categories that each participant reported experiencing in their first 18 years of life. Psychometric properties of both the ACE total score and the overall ACE module have been previously described (Ford et al., 2014).

#### 3.2.2. Outcome measures

High School Noncompletion: Participants categorized as not graduating high school were those responding that they had never attended school or only kindergarten, attended grades 1 through 8, or grades 9 through 11, when asked, “What is the highest grade or year of school you completed?” Only participants 25 years or older were included for this outcome.

Unemployment: Respondents were classified as unemployed if they indicated that they were “out of work for more than a year” or “out of work for less than a year” when asked about their current employment status. Participants who responded “Homemaker,” “student,” “retired,” or “unable to work” were excluded (*N* = 87).

Poverty Status: Household income was established by asking participants to respond “yes” or “no” to the question, “Is your annual household income from all sources:” less than different levels of income starting at $10,000 and increasing in intervals of $5000. Because BRFSS does not provide specific, individual-level income, poverty status was calculated by determining those at or below the 2010 federal poverty level using the 2010 guidelines provided by the U.S. Department of Health and Human Services (U.S. Department of Health and Human Services, 2010). In order to calculate poverty status we took the midpoint of the income range reported by participants and created a medium income for those in that range. That medium income was then divided by the number of adults and children reported in the household (for additional information on this method, see Hawaii Health Data Warehouse, 2016.).

### 3.3. Analytic procedure

Data analyses were conducted in R version 3.2 (R Core Team, 2013) and Mplus Version 7.0 (Muthén & Muthén, 1998–2012). We first examined simple frequency distributions for ACES and outcomes of interest; we then estimated the bivariate distributions of ACE exposure by several socio-demographic characteristics including age, sex, educational attainment, employment status, household income, and race/ethnicity. Logistic regression models were employed to adjust for the potential confounding effects of age, sex, and race/ethnicity on the relationship between the number of childhood exposures and high school noncompletion, and adjusted for age, sex, and race/ethnicity, and education for unemployment, and poverty status. Inspection of the inter-correlations among the predictor variables included in the models was performed to assess the potential for multicollinearity; no pairwise correlation exceeded 0.25 in magnitude. To test for dose-response relationships, we entered the number of childhood exposures as an ordinal variable (0, 1, 2, 3, 4 +) into a separate logistic regression model for each outcome.

## 4. Results

In the 10 states and D.C. sample, 40.7% reported no ACEs; 23.3% reported one ACE; 13.0% reported two ACEs; 7.8% reported three ACEs; and 15.1% reported four or more ACEs. The prevalence of specific ACEs varied from 5.9% (household member incarcerated) to 35.1% (emotionally abused by parent or adult in the household). Physical abuse was reported by 16.0% and sexual abuse was reported by 10.9%. As for our outcomes of interest, 6.0% of respondents had not graduated high school; 11.2% were currently unemployed; and, 22.4% lived in households at or below the federal poverty level. The distribution of ACE exposure by sample socio-demographic indicators is presented in Table 1.

### 4.1. ACEs and high school noncompletion, unemployment, and poverty status

In the logistic regression models (Table 2), the adjusted odds ratios were higher among individuals reporting high ACEs, suggesting greater risk for high school noncompletion, unemployment, and poverty. For example, compared to persons with no ACEs, persons with three ACEs were 1.53 times as likely not to graduate high school and 2.4 times as likely to be unemployed. Persons with four or more ACEs compared to those with no ACEs were 2.34 times as likely not to graduate high school, 2.3 times as likely to be unemployed, and 1.6 times as likely to live in a household reporting poverty. These findings are adjusted for age, sex, and race/ethnicity for the high school non-completion and, for age, sex, and race/ethnicity and education for both employment and income.

## 5. Discussion

### 5.1. Early adversity impacts life opportunities

This study examined the impact of ACEs on adult education, employment, and income. Findings reveal that ACEs were prevalent across women and minorities, with the exception of Asians. Those reporting four or more ACES were more likely to report high school noncompletion and household poverty. Both those reporting three ACES or four or more ACEs were more likely to report periods of unemployment. These findings are consistent with previous research (Liu et al., 2013; Anda et al., 2004; Font & Maguire-Jack, 2015).

### 5.2. Why this matters

The findings in this paper support our hypothesis that early experiences are related to later education, employment, and income. These outcomes of interest, in addition to their known impacts on health, are also important in terms of achieving multiple aspects of a meaningful life: education provides access to literacy, general and health-related knowledge, problem-solving skills, prestige, and influence over others and one’s own life; employment provides access to skills, prestige, and social influence; and, income provides access to material resources needed for health and living, as well as prestige (Braveman et al., 2005). But these outcomes are also more than the sum of their parts. Together, they are critical, interconnected components that confer access to life opportunities: education can lead to employment and employment leads to income. Lack of access to education, employment, and income affects not only individual and group health but also the ability of individuals and groups to achieve their full human potential, including fully participating as members of their communities and society.

Our study shows that the cumulative impact (four or more) of childhood adversity is associated with adult household poverty. Though there are limitations with our income measure (e.g., household vs. individual level), our study raises questions about the mainstream American narrative that views poverty primarily as the result of individual characteristics, including laziness, lack of intelligence, and/or lack of ambition without consideration of early childhood experiences or broader structural or institutional factors (Bullock, 2006; Lott, 2002). Perceptions and attitudes about people who are poor can begin as early as fourth grade (Woods, Kurtz-Costes, & Rowley, 2005). In addition to the burden of living with inadequate resources, people who are poor, including children, have the added burden of being blamed for their situations. Perceptions of social status can independently impact health (Marmot, 2004).

### 5.3. Early adversity reverberates across generations

The impact of early adversity is not only felt across one’s own life course. Researchers have previously documented the intergenerational continuity of child abuse and neglect (Merrick, Leeb, & Lee, 2013; Schofield, Lee, & Merrick, 2013). Lower educational attainment, higher unemployment, and lower household income also impact multiple generations. We know that the children of parents who are undereducated, underemployed, and/or living in poverty are themselves at heightened risk for poor educational outcomes that result in greater risk of unemployment and lower incomes (Tyler & Lofstom, 2009), demonstrating the potentially cyclical and intergenerational effects of these early adverse experiences. While the ideal of being able to move up the economic ladder during one’s lifetime and across generations is central to the American Dream, for several decades now, research shows that there are limitations to upward mobility in the United States (e.g., Fass, Dinan, & Aratani, 2009; Solon, 1992). Though almost two-thirds of Americans believe that those who are poor can rise up from the bottom (Gonchar, 2014), the fact is that the majority do not: in the U.S., 70% of those who are born in the bottom fifth never reach even the middle of the economic ladder; African American children born into poverty have an even greater risk of remaining in poverty as adults than white children born into poverty (The Pew Charitable Trusts, 2012). Cumulative adverse childhood experiences can increase the likelihood of adults living in poverty, which in turn can put their children at greater risk for remaining in poverty and experiencing lower attainment of life opportunities as adults, causing an intergenerational effect of these ACEs; this may be even more true for some racial/ethnic groups than others. These impacts on the children of adults who report early adversity are harsh enough. However, the impacts are likely to continue for their children when they become parents.

### 5.4. Preventing intergenerational early adversity and reduced life opportunities: Context matters

While all parents and caregivers may benefit from access to high-quality parenting programs (see, for example, Centers for Disease Control and Prevention, 2015), disrupting the intergenerational link between early adversity and diminished life opportunities will require moving beyond traditional parenting programs that focus on skills to address poor parenting to one that is focused on changing the contexts to assure safe, stable, nurturing relationships and environments for all children and their families. Preventing early adversity and its consequences for children, families, and communities will require an all-of-society approach. Providing support as early as possible to children experiencing adversity is critical to changing their life trajectory. For example, high-quality child care can buffer the consequences of adversity in the home (Watamura, Phillips, Morrissey, McCartney, & Bub, 2011). Adults who experienced early adversity also need support, including trauma-informed care and treatment, improvements in their immediate situations (e.g., food or housing insecurity), and structural changes that improve the opportunity for them and their children to achieve their full health and life potential.

Educational, service, and judicial systems that are trauma–informed and trauma–responsive can minimize the exacerbation of poor adult outcomes due to early adversity. School systems could consider other alternatives to suspending or expelling children to address behavioral problems that may well be the result of adverse childhood experiences. For example, a better understanding of ACEs and their impact on childhood behaviors has led some state legislatures (CT SB01053, 2015; CA AB420, 2014) and schools (Stevens, 2014) to ban suspending or expelling children for behavioral problems attributable to adverse childhood experiences. Government agencies or community-based organizations could facilitate families’ access to needed supports through the use of “one-stop shops” that automatically and simultaneously enroll families in all relevant services such as, for example, food, housing, healthcare and/or child support programs, as needed (Dorn, 2008). Judicial systems can minimize further traumatizing children by keeping juvenile offenders in the juvenile justice and correction systems and by providing evidence-based treatment (Guide to Community Preventive Services, 2015).

Services and programs are important, but developing long-term, sustainable solutions to poverty requires understanding and addressing structural barriers that contribute to and perpetuate intergenerational poverty and reduced life opportunities. It is important to also understand poverty as a far larger phenomenon than individual- or family-level income, including what it means to be poor in America. While increasing levels of education might lead to a decrease in risk for unemployment and poverty among individuals who are born into disadvantage (e.g., poverty or parental unemployment; see, for example, Heinrich & Holzer, 2011), quality education is not equitably available to all. For example, high poverty school districts receive about $1200 less funding per student than low poverty districts (Ushomirsky & Williams, 2015), which may be why schools with higher rates of low-income students have higher teacher turnover rates and a higher proportion of teachers not teaching in their area of certification (Orfield & Lee, 2005), larger classroom sizes (Condron & Roscigno, 2003), and poor facilities (Branham, 2004), among other impacts. The differences are even larger—about $2000 per student—between districts serving the most students of color and those serving the fewest (Ushomirsky & Williams, 2015). Black children also face additional barriers to education; according to an analysis by the U.S. Department of Education’s Office for Civil Rights, African American students represented 18% of students in the study sample, but 35% of students suspended once, 46% of those suspended more than once, and 39% of students expelled (U.S. Department of Education, 2012). Training teachers, school resource officers, administrators, and staff in trauma-informed practices (Stevens, 2014) and implicit bias (Devine, Forscher, Austin, & Cox, 2012) might reduce these discriminatory and negative disciplinary practices.

Education is critical given its connection to employment opportunities, but it is not equally protective. At all levels of educational attainment, African Americans and Latinos earn less than whites; African Americans and Latinos with a master’s degree have lifetime earnings that are lower than whites with a bachelor’s degree (Carnevale, Rose, & Cheah, 2011). Addressing the differential impact of education on income, including increased risk for poverty, will require understanding the contribution of structural determinants including, for example, macroeconomic policies. However, structural policies and processes that contribute to or inhibit moving from poverty are not limited to education; there is a clustering of challenges and disadvantages for people living in poverty that include reduced access to affordable housing (Turner et al., 2013), banking services (Burhouse et al., 2014), employment opportunities (Bertrand & Mullainathan, 2003; Pager, 2008), and increased risk for incarceration (Alexander, 2012). Policies that have the potential for changing conditions for children and families include policies that reduce poverty, especially concentrated poverty; policies that assure stable and affordable housing, access to high-quality and affordable child care and early education; and policies that assure access to health care, particularly mental health care for both children and parents (Fortson, Klevens, Merrick, Gilbert, & Alexander, 2016; Klevens, Barnett, Florence, & Moore, 2015). For example, a study of increases in state minimum wages showed that a modest increase of $1.00/h contributed to a decline in overall child abuse and neglect reports, including a 9.6% decrease in neglect reports (Raissian & Bullinger, 2017–in this issue).

### 5.5. Changing the narrative around early childhood adversity and life opportunities

Efforts to prevent early childhood adversity are largely informed by “narratives” (i.e., the way people think and talk about a problem and who is responsible for it) that attribute sole responsibility to parents. However, the challenges described here are beyond the ability of parents and families to solve on their own. The results of this study demonstrate that multiple early adverse experiences are associated with an increased likelihood of diminished life opportunities; it is clear from the literature that these diminished life opportunities can have lasting, generational effects. Therefore, to adequately understand and address the complex relationships between early adversity, health, and life opportunities requires expanding this narrative to include the multiple, interconnected structural policies and processes that place children and families at risk for poor outcomes across generations. Shaping a new narrative about the childhood roots of diminished adult life opportunities and the impact across generations includes creating an understanding that “making healthy choices” is simply not an option for some families and that more is needed to prevent childhood adversity. This new narrative can help guide our collective efforts to assure conditions for health for all people and may also help shift existing narratives around poverty and its causes such that these also consider the impact of structural policies and processes. One example of this approach can be found in the California Essentials for Childhood initiative where the Department of Public Health and the Department of Social Services’ Office of Child Abuse Prevention are collaborating to develop “a common agenda across multiple agencies and stakeholders to align activities, programs, policies, and funding so that all California children, youth, and their families have safe, stable, nurturing relationships and environments” (California Department of Public Health, 2015).

### 5.6. Future research

As noted throughout this paper, educational attainment, employment status, and income are frequently measured in child abuse and neglect studies but rarely as outcomes of interest. Investigations of key contributors to early adversity and their relative impact on outcomes are both scientifically important and necessary. However, it is also necessary to recognize that socio-demographic indicators are not self-assigned characteristics—individuals do not typically choose to have difficulty in school or problems in seeking employment—making these equally worthy of exploration as outcomes of interest. Our study is a simple demonstration of the impact of ACEs on common life opportunities and should not be considered a comprehensive examination. Rather, it is our hope to inspire other researchers to examine the impact of early adversity on life opportunities on a much broader scale. Expanding studies to include other important indicators of access to life opportunities including, for example, high-quality schools or living wages, and situating findings within a theoretical framework that broadens our understanding of them as more than attributes of individuals, is paramount to gaining a more accurate understanding of what is needed to protect all children from early adversity.

## 6. Limitations

There are a number of limitations with our analyses that should be considered. First, as a random digit dial survey, BRFSS 2010 excludes households without landlines but it uses post-stratification weights, which may partially correct for any bias caused by non-telephone coverage; these weights adjust for differences in probability of selection and nonresponse, as well as noncoverage. Also, ACEs are reported retrospectively; as such, memory of these events may be inaccurate. Additionally, child abuse and neglect and related items are sensitive topics and may be difficult or anxiety-provoking for some participants to report. States included in these analyses are not representative of the U.S., which limits generalizability of the findings. The types of adversity sampled by the BRFSS ACE module do not constitute the entire universe of early adversity that a child may experience and should therefore not be considered an exhaustive set. There are also limitations to our measure of income in that it reflects household versus individual-level income, as well as the fact that our measures of employment and income status at the time of the survey may not reflect employment and income status in the years preceding the survey. This study was cross-sectional and we therefore cannot infer causality, although childhood adversities clearly precede adult education, employment, and income and employment, and may therefore have an impact on the development of later opportunities. Also, the current study did not examine conditions such as access to supportive relationships and community resources (e.g., high-quality schools, good employment opportunities) that would likely buffer the effects of ACEs and subsequently improve life outcomes and opportunities.

## 7. Conclusion

In this study, we show that early adversity can negatively impact adult education, employment, and income. The importance of preventing early adversity has never been clearer given the numerous studies demonstrating adverse associations with subsequent health and life opportunities that reverberate across generations. Efforts to prevent early adversity, including child abuse and neglect, may be more successful if they broaden public and professional understanding of the links between early adversity and poverty and the structural barriers that reduce the likelihood of moving out of poverty. Understanding and addressing these impacts is critical for the full health and development of individuals, families, communities, and society.

## Figures and Tables

**Table 1:** Distribution of ACE exposure by sample socio-demographic characteristics.

	ACE Exposure				
	Zero Weighted M or % (95% CI)	One Weighted M or % (95% CI)	Two Weighted M or % (95% CI)	Three Weighted M or % (95% CI)	Four or more Weighted M or % (95% CI)
**Age** (Continuous)	45.92 (44.53, 45.51)	43.15 (42.52, 43.78)	42.92 (42.20, 43.65)	42.86 (41.95, 43.78)	40.67 (40.02, 41.31)
**Sex**					
Male	39.10 (37.30, 40.90)	24.73 (23.12, 26.35)	12.90 (11.71, 14.09)	8.62 (7.56, 9.67)	14.65 (13.29, 16.01)
Female	35.87 (34.31, 37.43)	22.87 (21.49, 24.24)	14.52 (13.34, 15.71)	8.39 (7.58, 9.21)	18.35 (17.12, 19.57)
**Race/Ethnicity**					
White	38.15 (36.83, 39.47)	24.26 (23.08, 25.45)	13.56 (12.65, 14.48)	8.48 (7.72, 9.25)	15.54 (14.54, 16.55)
Black or African American	27.78 (21.91, 33.65)	24.68 (19.27, 30.08)	14.48 (10.34, 18.64)	10.61 (7.21, 14.01)	22.46 (17.55, 27.37)
Hispanic	30.59 (25.80, 35.38)	20.47 (15.62, 25.32)	18.98 (13.62, 24.34)	7.68 (5.36, 9.99)	22.29 (17.73, 26.85)
Asian	59.53 (52.06, 67.00)	22.20 (15.86, 28.54)	10.12 (6.09, 14.15)	4.60 (2.38, 6.82)	3.56 (2.06, 5.06)
Other*	28.01 (22.75, 33.27)	19.07 (15.29, 22.86)	11.28 (8.71, 13.84)	10.71 (7.91, 13.59)	30.93 (26.01, 35.86)
**Education**					
Less than HS	28.56 (22.31, 34.80)	19.03 (13.89, 24.16)	12.40 (7.93, 16.87)	11.63 (6.73, 16.52)	28.39 (22.61, 34.17)
HS Graduate	34.48 (32.33, 36.74)	22.79 (20.79, 24.79)	13.35 (11.69, 15.01)	9.34 (7.81, 10.87)	20.04 (17.96, 22.11)
Some College	34.33 (32.15, 36.51)	23.91 (21.89, 25.94)	13.61 (12.13, 15.08)	8.99 (7.77, 10.21)	19.16 (17.33, 20.99)
College Graduate	42.47 (40.53, 44.41)	24.99 (23.26, 26.72)	13.94 (12.60, 15.29)	7.43 (6.50, 8.35)	11.17 (10.04, 12.30)
**Employment Status**					
Employed	39.14 (37.84, 40.43)	24.3 (23.16, 25.44)	13.75 (12.86, 14.65)	7.84 (7.18, 8.51)	14.96 (14.06, 15.86)
Unemployed	24.92 (21.77, 28.07)	20.41 (17.09, 23.74)	12.67 (10.05, 15.28)	14.16 (11.05, 17.26)	27.84 (23.80, 31.88)
**Household Income**					
Less than $10,000	23.98 (14.77, 33.20)	14.7 (9.23, 20.17)	20.01 (10.84, 29.17)	12.05 (4.23, 19.87)	29.26 (20.79, 37.73)
$10,000–$14,999	27.58 (19.82, 35.33)	18.56 (12.28, 24.84)	15.65 (8.86, 22.43)	8.76 (4.96, 12.57)	29.45 (22.06, 36.84)
$15,000–$19,999	32.19 (26.70, 37.68)	22.56 (16.94, 28.17)	12.48 (9.08, 15.88)	9.02 (5.60, 12.44)	23.75 (17.29, 30.21)
$20,000–$24,999	30.33 (26.02, 34.64)	21.03 (16.83, 25.23)	10.66 (8.22, 13.09)	10.55 (7.09, 14.02)	27.43 (22.92, 31.94)
$25,000–$34,999	35.14 (31.42, 38.85)	25.02 (20.76, 26.05)	12.68 (10.27, 15.09)	9.29 (7.17, 11.41)	17.87 (14.99, 20.76)
$35,000–$49,999	39.1 (36.09, 42.10)	23.40 (22.28, 27.09)	13.67 (11.69, 15.65)	8.77 (7.05, 10.48)	15.06 (13.01, 17.12)
$50,000–$74,999	37.83 (35.26, 40.41)	24.69 (22.28, 27.09)	13.59 (11.77, 15.41)	8.83 (7.48, 10.17)	15.06 (13.26, 16.87)
$75,000 or More	40.64 (38.65, 42.64)	24.8 (23.06, 26.54)	14.08 (12.66, 15.50)	7.52 (6.49, 8.56)	12.95 (11.58, 14.32)
N (Unweighted)	10,384	6481	3946	2540	4483

*Note*: Includes those who identified as American Indian or Alaskan Native only; Native Hawaiian or other Pacific Islander only; and, multiracial and/or other race.

**Table 2:** Adusted odds of high school noncompletion, unemployment, and household poverty status.

	Outcome		
	High School Noncompletion AOR (95% CI)^a^	Unemployment AOR (95% CI)^b^	Household Poverty Status AOR (95% CI)^b^
No ACEs (Reference)	1.00	1.00	1.00
One ACE	1.08 (0.87, 1.34)	1.25 (0.99, 1.57)	1.05 (0.65, 1.69)
Two ACEs	1.11 (0.85, 1.44)	1.35^⁎^ (1.04, 1.75)	1.57 (0.90, 2.74)
Three ACEs	1.53^⁎^ (1.09, 2.17)	2.39^⁎⁎⁎^ (1.80, 3.17)	1.25 (0.64, 2.43)
Four or more ACEs	2.34^⁎⁎⁎^ (1.85, 2.94)	2.31^⁎⁎⁎^ (1.83, 2.90)	1.56^⁎^ (1.01, 2.42)

⁎ *p <* 0.05,

⁎⁎⁎ *p* < 0.001.

a Odds ratios are adjusted for age, race, and sex.

b Odds ratios are adjusted for age, race, sex, and educational attainment.

## Appendix A

The CSDH framework draws on many models that preceded it, but provides needed specificity to inform in-depth explorations of the mechanisms and pathways through which structural policies and processes contribute to differential exposure, differential vulnerability, and, consequently, differential health outcomes.

Briefly, the main domains of the CSDH framework include:

- ***Structural Determinants: Socioeconomic Political Context*.** The structural, cultural, and functional policies and processes that shape how societies are organized—governance structures, macroeconomic policies, social policies, etc.
- ***Structural Determinants: Socioeconomic Position*.** This domain describes how structural policies and processes interact to effectively assign socioeconomic position based on social characteristics (e.g., race/ethnicity, gender) through more or less access to essential resources including education, occupation, and income.
- ***Intermediary Determinants*.** Broadly encompassing living and working conditions, this domain also includes psychosocial, behavioral and biological characteristics, as well as the health system.
- ***Cross-Cutting Determinants** (e.g., social capital and social cohesion)*. This domain acknowledges human agency and the role of people in the shaping of policies and processes that effectively determine how societies are organized.
- ***Health Equity*:** The comparison of the health of populations based on hierarchies of social advantage and disadvantage (e.g., race/ethnicity, income, gender).

## Abbreviations:

ACEs: Adverse Childhood Experiences
BRFSS: Behavioral Risk Factor Surveillance System
CDC: Centers for Disease Control & Prevention
CSDH: Commission on the Social Determinants of Health

## References

[R1] Alexander M (2012). The new Jim Crow: Mass incarceration in the age of color blindness New York: New Press.

[R2] Anda RF, Fleisher VI, Felitti VJ, Edwards VJ, Whitfield CL, Dube SR, & Williamson DF (2004). Childhood abuse, household dysfunction, and indicators of impaired adult worker performance. The Permanente Journal, 8(1), 30–38. 26704603 10.7812/tpp/03-089 PMC4690705

[R3] Anda RF, Whitfield CL, Felitti VJ, Chapman D, Edwards VJ, Dube SR, & Williamson DF (2002). Adverse childhood experiences, alcoholic parents, and later risk of alcoholism and depression. Psychiatriatry Services, 53(8), 1001–1009. 10.1176/appi.ps.53.8.1001 12161676

[R4] Andersen JP, & Blosnich J (2013). Disparities in adverse childhood experiences among sexual minority and heterosexual adults: Results from a multi-state probability-based sample. PloS One, 8(1), e54691. 10.1371/journal.pone.0054691. 23372755 PMC3553068

[R5] Berkman LF, Kawachi I, & Glymour M (2014). Social Epidemiology (Ed.). New York, NY: Oxford University Press.

[R6] Bertrand M, & Mullainathan S (2003). Are Emily and Greg more employable than Lakisha and Jamal? A field experiment on labor market discrimination. National Bureau of Economics Research Working Paper No. 9873 (Retrieved from http://www.nber.org/papers/w9873).

[R7] Bierhaus A, Wolf J, Andrassy M, Rohleder N, Humpert PM, Petrov D, … Nawroth PP (2003). A mechanism converting psychosocial stress into mononuclear cell activation. Proceedings of the National Academy of Sciences of the United States of America, 100(4), 1920–1925. 12578963 10.1073/pnas.0438019100 PMC149934

[R8] Branham D (2004). The wise man builds his house upon the rock: The effects of inadequate school building infrastructure on student attendance. Social Science Quarterly, 85(5), 1112–1128. 10.1111/j.0038-4941.2004.00266.x.

[R9] Braveman P (2014). What are health disparities and health equity? We need to be clear. Public Health Reports, 129(S2), 5–8. 10.2105/AJPH.2009.166082. PMC3863701 24385658

[R10] Braveman PA, Cubbin C, Egerter S, Chideya D, Marchi KS, Metzler M, & Posner S (2005). Socioeconomic status in health research: One size does not fit all. Journal of the American Medical Association, 294(22), 2879–2888. 10.1001/jama.294.22.2879. 16352796

[R11] Braveman PA, Cubbin C, Egerter S, Williams DR, & Pamuk E (2010). Socioeconomic disparities in health in the United States: What the patterns tell us. American Journal of Public Health, 100(Suppl. 1), S186–S196. 10.2105/AJPH.2009.166082. 20147693 PMC2837459

[R12] Brown DW, Anda RF, Felitti VJ, Edwards VJ, Malarcher AM, Croft JB, & Giles WH (2010). Adverse childhood experiences are associated with the risk of lung cancer: A prospective cohort study. BMC Public Health, 10, 20. 10.1186/1471-2458-10-20. 20085623 PMC2826284

[R13] Brown DW, Anda RF, Tiemeier H, Felitti VJ, Edwards VJ, Croft JB, & Giles WH (2009). Adverse childhood experiences and the risk of premature mortality. American Journal of Preventive Medicine, 37(5), 389–396. 10.1016/j.amepre.2009.06.021. 19840693

[R14] Bullock H (2006). Justifying inequality: A social psychological analysis of beliefs about poverty and the poor. National Poverty Center Working Paper Series #06–08 (Retrieved from http://141.211.144.109/publications/workingpaper06/paper08/working_paper06-08.pdf).

[R15] Burhouse S, Chu S, Goodstein R, Northwood J, Osaki Y, & Sharma D (2014, October). 2013 FDIC National Survey of Unbanked and Underbanked Households Retrieved from https://www.fdic.gov/householdsurvey/2013report.pdf

[R16] CA AB420 | 2013–2014 | Pupil discipline: Suspensions and expulsions: Willful defiance. Regular Session 2014 September 27 (LegiScan. Retrieved November 04, 2015, from https://legiscan.com/CA/bill/AB420/2013)

[R17] California Department of Public Health (2015). Essentials for Childhood Initiative: Safe, Stable, Nurturing Relationships and Environments Retrieved from http://www.cdph.ca.gov/programs/Pages/ChildMaltreatmentPrevention.aspx

[R18] Carnevale AP, Rose SJ, & Cheah B (2011). The college payoff: Education, occupations, lifetime earnings. The Georgetown University Center on Education and the Workforce Retrieved from https://repository.library.georgetown.edu/bitstream/handle/10822/559300/collegepayoff-complete.pdf?sequence=1&isAllowed=y

[R19] Centers for Disease Control and Prevention (2010). Adverse childhood experiences reported by adults in 5 states. Morbidity & Mortality Weekly Report, 59(49), 1609–1613 (Retrieved from http://www.cdc.gov/mmwr/preview/mmwrhtml/mm5949a1.htm). 21160456

[R20] Centers for Disease Control and Prevention (2015). Essentials for parenting toddlers and preschoolers Retrieved from http://www.cdc.gov/parents/essentials/index.html

[R21] Chapman DP, Whitfield CL, Felitti VJ, Dube SR, Edwards VJ, & Anda RF (2004). Adverse childhood experiences and the risk of depressive disorders in adulthood. Journal of Affective Disorders, 82(2), 217–225. 10.1016/j.jad.2003.12.013. 15488250

[R22] Colborn T (2004). Neurodevelopment and endocrine disruption. Environmental Health Perspectives, 112(9), 944–949. 10.1289/ehp.6601. 15198913 PMC1247186

[R23] Condron DJ, & Roscigno VJ (2003). Disparities within: Unequal spending and achievement in an urban school district. Sociology of Education, 76(1), 18–36. 10.2307/3090259.

[R24] Covey HC, Menard S, & Franzese RJ (2013). Effects of adolescent physical abuse, exposure to neighborhood violence, and witnessing parental violence on adult socioeconomic status. Child Maltreatment, 18(2), 85–97. 10.1177/1077559513477914. 23420296

[R25] CT SB01053 (2015). General Assembly June 23, 2015 LegiScan. Retrieved November 04, 2015, from https://legiscan.com/CT/bill/SB01053/2015

[R26] Currie J, & Widom CS (2010). Long-term consequences of child abuse and neglect on adult economic well-being. Child Abuse and Neglect, 15(2), 111–120. 10.1177/1077559509355316. PMC3571659 20425881

[R27] Danese A, Moffitt TE, Pariante CM, Ambler A, Poulton R, & Caspi A (2008). Elevated inflammation levels in depressed adults with a history of childhood maltreatment. Archives of General Psychiatry, 65(4), 409–415. 10.1001/archpsyc.65.4.409. 18391129 PMC2923056

[R28] Devine PG, Forscher PS, Austin AJ, & Cox WT (2012). Long-term reduction in implicit race bias: A prejudice habit-breaking intervention. Journal of Experimental Social Psychology, 48. 10.1016/j.jesp.2012.06.003. PMC3603687 23524616

[R29] Dorn S (2008). How policy makers could use automation to help families and children First Focus. Big ideas for children (Retrieved from http://firstfocus.org/resources/report/big-ideas-investing-nations-future/).

[R30] Dube SR, Anda RF, Felitti VJ, Chapman DP, Williamson DF, & Giles WH (2001). Childhood abuse, household dysfunction, and the risk of attempted suicide throughout the life span: Findings from the Adverse Childhood Experiences Study. Journal of the American Medical Association, 286(24), 3089–3096. 10.1001/jama.286.24.3089. 11754674

[R31] Dube SR, Anda RF, Felitti VJ, Edwards VJ, & Croft JB (2002). Adverse childhood experiences and personal alcohol abuse as an adult. Addictive Behaviors, 27(5), 713–725. 10.1016/S0306-4603(01)00204-0. 12201379

[R32] Dube SR, Felitti VJ, Dong M, Chapman DP, Giles WH, & Anda RF (2003). Childhood abuse, neglect, and household dysfunction and the risk of illicit drug use: The Adverse Childhood Experiences Study. Pediatrics, 111(3), 564–572. 10.1542/peds.111.3.564. 12612237

[R33] Fang X, Brown DS, Florence CS, & Mercy JA (2012). The economic burden of child maltreatment in the United States and implications for prevention. Child Abuse & Neglect, 36(2), 156–165. 10.1016/j.chiabu.2011.10.006. 22300910 PMC3776454

[R34] Fass S, Dinan KA, & Aratani Y (2009). Child poverty and intergenerational mobility The National Center for Children in Poverty (http://www.nccp.org/publications/pdf/text_911.pdf).

[R35] Felitti VJ, Anda RF, Nordenberg D, Williamson DF, Spitz AM, Edwards V, & Marks JS (1998). Relationship of childhood abuse and household dysfunction to many of the leading causes of death in adults: The Adverse Childhood Experiences (ACE) Study. American Journal of Preventive Medicine, 14(4), 245–258. 10.1016/s0749-3797(98)00017-8. 9635069

[R36] Finkelhor D, Shattuck A, Turner H, & Hamby S (2013). Improving the adverse childhood experiences study scale. JAMA Pediatrics, 167(1), 70–75. 10.1001/jamapediatrics.2013.420. 23403625

[R37] Flaherty EG, Thompson R, Dubowitz H, Harvey EM, English DJ, Proctor LJ, & Runyan DK (2013). Adverse childhood experiences and child health in early adolescence. JAMA Pediatrics, 167(7), 622–629. 10.1001/jamapediatrics.2013.22. 23645114 PMC3732117

[R38] Flaherty EG, Thompson R, Litrownik AJ, Theodore A, English DJ, Black MM, … Dubowitz H (2006). Effect of early childhood adversity on child health. Archives of Pediatrics & Adolescent Medicine, 160(12), 1232–1238. 10.1001/archpedi.160.12.1232. 17146020

[R39] Font SA, & Maguire-Jack K (2015). Pathways from childhood abuse and other adversities to adult health risks: The role of adult socioeconomic conditions. Child Abuse & Neglect, 51, 390–399. 10.1016/j.chiabu.2015.05.013. 26059537 PMC4670808

[R40] Ford ES, Anda RF, Edwards VJ, Perry GS, Zhao G, Li C, & Croft JB (2001). Adverse childhood experiences and smoking status in five states. Preventive Medicine, 53(3), 188–193. 10.1016/j.ypmed.2011.06.015. 21726575

[R41] Ford DC, Merrick MT, Parks SE, Breiding SE, Gilbert MJ, Edwards VJ, … Thompson WW (2014). Examination of the factorial structure of adverse childhood experiences and recommendations for three subscale scores. Psychology of Violence, 4, 432–444. 10.1037/a0037723. 26430532 PMC4587306

[R42] Fortson BL, Klevens J, Merrick MT, Gilbert LK, & Alexander SP (2016). Preventing child abuse and neglect: A technical package for policy, norm, and programmatic activities Atlanta, GA: National Center for Injury Prevention and Control, Centers for Disease Control and Prevention (http://www.cdc.gov/violenceprevention/pdf/can-prevention-technical-package.pdf).

[R43] Fox BH, Perez N, Cass E, Baglivio MT, & Epps N (2015). Trauma changes everything: Examining the relationship between adverse childhood experiences and serious, violent and chronic juvenile offenders. Child Abuse & Neglect, 46, 163–173. 10.1016/j.chiabu.2015.01.011. 25703485

[R44] Gilbert LK, Breiding MJ, Merrick MT, Thompson WW, Ford DC, Dhingra SS, & Parks SE (2010). Childhood adversity and adult chronic disease: An update from ten states and the District of Columbia. American Journal of Preventive Medicine, 48(3), 345–349. 10.1016/j.amepre.2014.09.006. 25300735

[R45] Gonchar M (2014, December 12). Is It Possible to Start Out Poor in This Country, Work Hard and Become Well-Off? New York Times Retrieved December 15, 2015 from http://learning.blogs.nytimes.com/2014/12/12/is-it-possible-to-start-out-poor-in-this-country-work-hard-and-become-well-off/?_r=0

[R46] Guide to Community Preventive Services. (2015). Violence prevention: Youth transfer to adult criminal system Retrieved from www.thecommunityguide.org/violence/youth.html

[R47] Hawaii Health Data Warehouse (2016). HHDW poverty level Retrieved from http://www.hhdw.org/wp-content/uploads/BRFSS-Poverty-Level-Methodology.pdf

[R48] Healthy People 2020. Retrieved from http://www.healthypeople.gov/2020/About-Healthy-People

[R49] Heinrich CJ, & Holzer HJ (2011). Improving education and employment for disadvantaged young men: Proven and promising strategies. The Annals of the American Academy of Political and Social Science, 635, 163–191. 10.1177/0002716210391968.

[R50] Hillis SD, Anda RF, Felitti VJ, & Marchbanks PA (2001). Adverse childhood experiences and sexual risk behaviors in women: A retrospective cohort study. Family Planning Perspectives, 33(5), 206–211. 10.2307/2673783. 11589541

[R51] Kaplan GA, Pamuk ER, Lynch JW, Cohen RD, & Balfour JL (1996). Inequality in income and mortality in the United States: analysis of mortality and potential pathways. British Medical Journal, 312(7037), 999–1003. 10.1136/bmj.312.7037.999. 8616393 PMC2350835

[R52] Klevens J, Barnett SB, Florence C, & Moore D (2015). Exploring policies for the reduction of child physical abuse and neglect. Child Abuse & Neglect, 40, 1–11. 10.1016/j.chiabu.2014.07.013. 25124051 PMC4689429

[R53] Krieger N (2001). Theories for social epidemiology in the 21st century: An ecosocial perspective. International Journal of Epidemiology, 30(4), 668–677. 10.1093/ije/30.4.668. 11511581

[R54] Last JM (Ed.). (2007). A dictionary of public health New York: Oxford University Press.

[R55] Liu Y, Croft JB, Chapman DP, (2013). Relationship between adverse childhood experiences and unemployment among adults from five U.S. states. Social Psychiatry and Psychiatric Epidemiology, 48(3), 357–369. 10.1007/s00127-012-0554-1. 22869349 PMC4539022

[R56] Lott B (2002). Cognitive and behavioral distancing from the poor. American Psychologist, 57(2), 100–110. 10.1037/0003-066X.57.2.100. 11899553

[R57] Macmillan R, & Hagan J (2004). Violence in the transition to adulthood: Adolescent victimization, education, and socioeconomic attainment in later life. Journal of Research on Adolescence, 14(2), 127–158. 10.1111/j.1532-7795.2004.01402001.x.

[R58] Marmot M (2004). The Status Syndrome: How Social Standing Affects Our Health and Longevity New York, New York: Henry Holt and Company.

[R59] McCrory E, De Brito SA, & Viding E (2011). The impact of childhood maltreatment: A review of neurobiological and genetic factors. Frontiers in Psychiatry, 2, 48. 10.3389/fpsyt.2011.00048. 21847382 PMC3148713

[R60] Merrick MT, Leeb RT, & Lee RD (2013). Examining the role of safe, stable, and nurturing relationships in the intergenerational continuity of maltreatment—introduction to the special issue. Journal of Adolescent Health, 53(5), S1–S3. 10.1016/j.jadohealth.2013.06.017. 24059933

[R61] Muthén LK, & Muthén BO (1998–2012). Mplus User’s Guide (7th ed.). Los Angeles, CA: Muthén & Muthén.

[R62] Orfield G, & Lee C (2005). Why segregation matters: Poverty and educational inequality The Civil Rights Project: Harvard University (Retrieved December 15, 2015 from http://bsdweb.bsdvt.org/district/EquityExcellence/Research/Why_Segreg_Matters.pdf).

[R63] Pager D (2008). Marked: Race, crime, and finding work in an era of mass incarceration Chicago & London: The University of Chicago Press.

[R64] R Core Team (2013). R: A language and environment for statistical computing Vienna, Austria: R Foundation for Statistical Computing (Retrieved from http://www.R-project.org).

[R65] Raissian KM, & Bullinger LR (2017). Money matters: Does the minimum wage affect child maltreatment rates? Children and Youth Services Review, 72, 60–70 (in this issue).

[R66] Roth TL, Lubin FD, Funk AJ, & Sweatt JD (2009). Lasting epigenetic influence of early-life adversity on the BDNF gene. Biological Psychiatry, 65(9), 760–769. 10.1016/j.biopsych.2008.11.028. 19150054 PMC3056389

[R67] Sansone RA, Leung JS, & Wiederman MW (2012). Five forms of childhood trauma: Relationships with employment in adulthood. Child Abuse & Neglect, 36(9), 676–679. 10.1016/j.chiabu.2012.07.007. 22954641

[R68] Schofield TJ, Lee RD, & Merrick MT (2013). Safe, stable, nurturing relationships as a moderator of intergenerational continuity of child abuse and neglect: A meta-analysis. Journal of Adolescent Health, 53(5), S32–S38. 10.1016/j.jadohealth.2013.05.004. PMC3784834 24059937

[R69] Shonkoff JP, & Garner AS (2012). The lifelong effects of early childhood adversity and toxic stress. Pediatrics, 129(1), e232–e246. 10.1542/peds.2011-2663. 22201156

[R70] Shonkoff JP, Boyce WT, & McEwen BS (2009). Neuroscience, molecular biology, and the childhood roots of health disparities: Building a new framework for health promotion and disease prevention. Journal of the American Medical Association, 301(21), 2252–2259. 10.1001/jama.2009.754. 19491187

[R71] Smith GD, & Egger M (1996). Commentary: understanding it all—health, metatheories, and mortality trends. British Medical Journal, 313(7072), 1584–1585. 10.1136/bmj.313.7072.1584. 8990993 PMC2359097

[R72] Solar O, & Irwin A (2010). A conceptual framework for action on the social determinants of health. Social Determinants of Health Discussion Paper 2 (Policy and Practice). Retrieved from http://apps.who.int/iris/bitstream/10665/44489/1/9789241500852_eng.pdf

[R73] Solon G (1992). Intergenerational income mobility in the United States. American Economic Review, 82(3), 393–408 (Retrieved from http://www.jstor.org/stable/2117312?seq=1#page_scan_tab_contents).

[R74] Stevens JA (2014). Children’s resilience initiative in Walla Walla, WA, draws spotlight to trauma-sensitive school. ACES Too High News (Retrieved December 3, 2015 from http://acestoohigh.com/2014/10/07/childrens-resilience-initiative-in-walla-walla-wa-draws-spotlight-to-trauma-sensitive-school/).

[R75] Szyf M (2009). The early life environment and the epigenome. Biochimica et Biophysica Acta, 1790(9), 878–885. 10.1016/j.bbagen.2009.01.009. 19364482

[R76] The Pew Charitable Trusts (2012 July). Pursuing the American Dream: Economic Mobility Across Generations (Retrieved from http://www.pewtrusts.org/~/media/legacy/uploadedfiles/pcs_assets/2012/PursuingAmericanDreampdf.pdf).

[R77] Turner MA, Santos R, Levy DK, Wissoker D, Aranda C, & Pitingolo R (2013). Housing Discrimination against Racial and Ethnic Minorities 2012: Executive Summary Washington, DC: Urban Institute (Retrieved fromhttp://www.huduser.gov/portal/Publications/pdf/HUD-514_HDS2012_execsumm.pdf).

[R78] Tyler JH, & Lofstom M (2009). Finishing high school: Alternative pathways and drop-out recovery. Future of Children, 19, 77–103. 10.1353/foc.0.0019. 21141706

[R79] U.S. Department of Education. Office of Civil Rights. Revealing New Truths About Our Nation’s Schools, 2012 March 12. (Retrieved from http://www2.ed.gov/about/offices/list/ocr/docs/crdc-2012-data-summary.pdf)

[R80] U.S. Department of Health and Human Services (2010). Delayed update of the HHS poverty guidelines for the remainder of 2010. Federal Register 75, no. 148, 45628–45629.

[R81] Ushomirsky N, & Williams D (2015). The Funding Gap. The Education Trust (Retrieved from http://edtrust.org/wp-content/uploads/2014/09/FundingGaps2015_TheEducationTrust1.pdf).

[R82] Watamura SE, Phillips DA, Morrissey TW, McCartney K, & Bub K (2011). Double jeopardy: poorer social-emotional outcomes for children in the NICHD SECCYD experiencing home and child-care environments that confer risk. Child Development, 82(1), 48–65. 10.1111/j.1467-8624.2010.01540.x. 21291428

[R83] Whitfield CL, Anda RF, Dube SR, & Felitti VJ (2003). Violent childhood experiences and the risk of intimate partner violence in adults: Assessment in a large health maintenance organization. Journal of Interpersonal Violence, 18(2), 166–185. 10.1177/0886260502238733.

[R84] Wilkinson RG, & Pickett KE (2006). Income inequality and population health: A review and explanation of the evidence. Social Science & Medicine, 62(7), 1768–1784. 10.1016/j.socscimed.2005.08.036. 16226363

[R85] Woods TA, Kurtz-Costes B, & Rowley SJ (2005). The development of stereotypes about the rich and poor: Age, race, and family income differences in beliefs. Journal of Youth and Adolescence, 34(5), 437–445. 10.1007/s10964-005-7261-0.

[R86] Ye D, & Reyes-Salvail F (2014). Adverse childhood experiences among Hawai’i adults: Findings from the 2010 Behavioral Risk Factor Survey. Hawai’i Journal of Medicine & Public Health, 73(6), 181–190 (Retrieved from http://www.ncbi.nlm.nih.gov/pmc/articles/PMC4064343/). 24959392 PMC4064343

[R87] Zielinski DS (2009). Child abuse and neglect and adult socioeconomic well-being. Child Abuse & Neglect, 33, 666–678. 10.1016/j.chiabu.2009.09.001. 19811826

